# Ibogaine administration following repeated morphine administration upregulates myelination markers 2′, 3′-cyclic nucleotide 3′-phosphodiesterase (CNP) and myelin basic protein (MBP) mRNA and protein expression in the internal capsule of Sprague Dawley rats

**DOI:** 10.3389/fnins.2024.1378841

**Published:** 2024-07-24

**Authors:** Demi Govender, Leila Moloko, Maria Papathanasopoulos, Nancy Tumba, Gavin Owen, Tanya Calvey

**Affiliations:** ^1^School of Anatomical Sciences, Faculty of Health Sciences, University of the Witwatersrand, Johannesburg, South Africa; ^2^Department of Human Biology, Faculty of Health Sciences, University of Cape Town, Cape Town, South Africa; ^3^HIV Pathogenesis Research Unit, Faculty of Health Sciences, University of the Witwatersrand, Johannesburg, South Africa; ^4^Neuroscience Institute, University of Cape Town, Cape Town, South Africa

**Keywords:** ibogaine, psychedelic medicine, white matter, opioid use disorder, oligodendrocytes

## Abstract

Ibogaine is a psychedelic alkaloid being investigated as a possible treatment for opioid use disorder. Ibogaine has a multi-receptor profile with affinities for mu and kappa opioid as well as NMDA receptors amongst others. Due to the sparsity of research into ibogaine's effects on white matter integrity and given the growing evidence that opioid use disorder is characterized by white matter pathology, we set out to investigate ibogaine's effects on two markers of myelination, 2′, 3′-cyclic nucleotide 3′-phosphodiesterase (CNP) and myelin basic protein (MBP). Fifty Sprague Dawley rats were randomly assigned to five experimental groups of *n* = 10; (1) a saline control group received daily saline injections for 10 days, (2) a morphine control group received escalating morphine doses from 5 to 15 mg/kg over 10 days, (3) an ibogaine control group that received 10 days of saline followed by 50 mg/kg ibogaine hydrochloride, (4) a combination morphine and ibogaine group 1 that received the escalating morphine regime followed by 50 mg/kg ibogaine hydrochloride and (5) a second combination morphine and ibogaine group 2 which followed the same morphine and ibogaine regimen yet was terminated 72 h after administration compared to 24 h in the other groups. White matter from the internal capsule was dissected and qPCR and western blotting determined protein and gene expression of CNP and MBP. Morphine upregulated CNPase whereas ibogaine alone had no effect on CNP mRNA or protein expression. However, ibogaine administration following repeated morphine administration had an immediate effect by increasing CNP mRNA expression. This effect diminished after 72 h and resulted in a highly significant upregulation of CNPase protein at 72 h post administration. Ibogaine administration alone significantly upregulated protein expression yet downregulated MBP mRNA expression. Ibogaine administration following repeated morphine administration significantly upregulated MBP mRNA expression which increased at 72 h post administration resulting in a highly significant upregulation of MBP protein expression at 72 h post administration. These findings indicate that ibogaine is able to upregulate genes and proteins involved in the process of remyelination following opioid use and highlights an important mechanism of action of ibogaine's ability to treat substance use disorders.

## 1 Introduction

Opioids continue to be the group of substances with the highest contribution to severe drug-related harm, including fatal overdoses (United Nations Office on Drugs and Crime, 2023). An estimated 60 million people engaged in non-medical opioid use in 2021. Opioids remain the leading cause of deaths from fatal overdoses. Opioids accounted for nearly 70% of the 128,000 deaths attributed to drug use disorders in 2019. Opioid use disorders (OUDs) also accounted for the majority (71% of the 18 million healthy years) of life lost owing to premature death and disability in 2019 (United Nations Office on Drugs and Crime, 2023).

Ibogaine is the primary alkaloid in *Tabernathe iboga* root bark and may have anti-addictive effects that lead to reduced drug cravings, decreased symptoms of withdrawal, and prevention of relapse (Maisonneuve et al., 1991; Sheppard, 1994; Alper et al., 1999; Mash et al., 2000, 2018; Parker and Siegel, 2001; Schenberg et al., 2014; Barsuglia et al., 2018; Calvey and Howells, 2018). Ibogaine undergoes extensive first-pass metabolism to noribogaine (the principle metabolite) by cytochrome P4502D6 in the gut wall and liver following oral administration. Ibogaine is cleared from the blood within 24 h (t1/2 = 4–6 h) depending on CYP2D6 genotype. Noribogaine, on the other hand, is eliminated over 5–7 days (t1/2 = 24–30 h; Mash, 2023).

The mechanisms of action of ibogaine continue to be explored but likely result from its multiple receptor affinities and polypharmacology. Ibogaine inhibits transport of serotonin and dopamine and is a serotonin reuptake blocker. It is a non-competitive inhibitor on nicotinic receptors such as the ganglionic and alpha-3 beta-4 subtype, a partial agonist of mu and kappa opioid receptors, and an NMDA channel blocker (Mash, 2023). Noribogaine binds to the serotonin transporter with a higher affinity (Villalba et al., 2024) and is a weak mu opioid receptor antagonist. Ibogaine is able to produce a neuroadaptive effect on endogenous opioid systems which reverses opioid tolerance (Barsuglia et al., 2018; Calvey and Howells, 2018; Corkery, 2018). Both ibogaine and noribogaine modulate the analgesic effect and physical tolerance to morphine (Calvey and Howells, 2018; Mash, 2023). Further, ibogaine possesses an opiate replacement mechanism of action as reported for compounds such as methadone possibly due to its agonism on the mu opioid receptor, however, neither ibogaine nor noribogaine produce signs and symptoms of opioid intoxication in opioid naïve persons (Mash et al., 1995; Zubaran et al., 1999; Baumann et al., 2001a,b; Barsuglia et al., 2018; Corkery, 2018).

We have conducted research on mechanisms of action of ibogaine for several years. Findings from our lab indicate that ibogaine HCl affects gene and protein expression in proteins related to substance use disorders. For example, ibogaine HCl downregulates the glutamate ionotropic receptor AMPA subunit 1 (GRIA1; Calvey et al., 2019a,b) and upregulates histone deacetylase 2,3 (HDAC2,3) which is involved in epigenetic mechanisms diminishing opioid tolerance (Moloko et al., 2019).

Of particular interest is the role of white matter on the pathology of SUDs and opioid addiction. Oligodendrocytes have been understudied with regards to drug abuse and its effects (Miguel-Hidalgo, 2018) but the effect of opioid addiction is associated with demyelination (Liu et al., 2013; Fan et al., 2018). Upadhyay et al. (2010) showed decreased anisotropy of white matter tracts specifically in the amygdala-specific tracts which suggests decreased white matter tract connectivity in brains affected by opioid addiction. This further suggests that opioid addiction causes demyelination. There are two myelin markers that have commonly been used to assess the amount of myelin present in a sample: myelin basic protein (MBP) and 2′, 3′-cyclic nucleotide 3′-phosphodiesterase (CNP) protein. Changes in expression and abundance of these proteins can be used as early markers for increased or decreased myelin (Lindner et al., 2008; Oberoi et al., 2019).

Myelin basic protein (MBP) is the second most abundant myelin sheath protein and has four main isoforms which are 21.5, 18.5, 17, and 14 kDa (Akiyama et al., 2002). It compromises 30% of the total myelin protein and is a structural protein that is essential for CNS myelin (Boggs, 2006). It is bound to the cytosolic membrane of the oligodendrocytes between the layers of cells. It is involved in the adhesion of the cytosolic surface of the myelin sheath layers (De Vries et al., 1997; Boggs, 2006). Therefore, its main function is adhesion for the myelin sheath formation and has a critical role in myelination or remyelination. CNPase is an enzyme and structural protein present in the cytoplasm of oligodendrocytes that catalyzes the hydrolysis of 2′,3′-cyclic nucleotides (Verrier et al., 2013). It is utilized during the early stages of oligodendrocyte differentiation and has been associated with compacting the myelin layers on axons as it is found on the outer layers of the cells (De Vries et al., 1997; Maier et al., 2008).

Very little is known about ibogaine's effect on white matter or its role in de- or remyelination. The pharmacology of ibogaine and noribogaine suggests potential to increase white matter. Ibogaine increases BDNF and GDNF, which has been shown to have neuroplastic effects in addiction (Carnicella and Ron, 2009; Marton et al., 2019). Ibogaine and noribogaine are partial kappa opioid receptor agonists (Glick et al., 1997; Glick and Maisonneuve, 1998; Alper, 2001; Maillet et al., 2015; Mash, 2023). The kappa opioid receptor has been shown to influence remyelination through oligodendrocytes (Du et al., 2016; Mei et al., 2016). The kappa opioid receptor is an important regulator of oligodendrocyte differentiation and remyelination. This regulation is achieved by controlling the oligodendrocyte precursor cells (OPCs) developing into oligodendrocytes (Du et al., 2016; Mei et al., 2016). The kappa opioid receptor is expressed on OPCs and in white matter such as the corpus callosum (Mei et al., 2016). Of interest is ibogaine's dual affinity for mu and kappa opioid receptors. Several dual kappa/mu opioid receptor agonists have produced strong antinociception effects in mice while lacking the typical dysphoric or addictive properties of pure kappa- or pure mu opioid receptor agonists, respectively (Khan et al., 2022). This highlights the inter-play between the functions of these two types of opioid receptors. Very little is known about this relationship in ibogaine or in the process of myelination.

We were, therefore, interested in observing ibogaine's effect on myelination markers. Preliminary findings indicated that ibogaine administration upregulated CNPase protein expression in rats (Govender et al., 2020). We expanded the study to include analysis of both protein and gene expression of CNPase and MBP in groups of Sprague Dawley rats. The experiment was designed to indicate effects of ibogaine and morphine administration as well as the temporal mechanisms of remyelination.

## 2 Materials and methods

### 2.1 Animal experiment

The ethical approval for this study was issued by the Animal Research Ethics Committee at the University of the Witwatersrand. Fifty male Sprague-Dawley (SD) rats, post-natal days 42–45, were on a 12-h light/dark cycle with food and water access as needed. All the animals were allowed a pre-treatment acclimatization period of 1 week. Two rats were housed in 1500U Techniplast Eurostandard Type IV S cages to prevent social isolation. Animals were assigned randomly to one of five groups and weighed daily, to calculate accurate dosages of ibogaine, morphine and saline. The animals were randomly divided into five groups of 10 rats: saline control, ibogaine only, morphine only, morphine ibogaine 1 test group and morphine ibogaine 2 test group with a 3-day waiting period before termination.

The saline control group (*n* = 10) received daily 1 ml/kg subcutaneous (s.c.) saline (0.9% sodium chloride, Adcocare, Adcock Ingram Critical Care) for 10 days. The rats were terminated 24 h following the last administration.

The morphine group (*n* = 10) was administered daily morphine (s.c.) for 10 days according to an escalated morphine protocol starting at 5 mg/kg for 48 h, 7.5 mg/kg for 72 h, 10 mg/kg for 72 h, and 15 mg/kg for 48 h. The rats were terminated 24 h following the last administration.

The ibogaine group (*n* = 10) received daily saline (0.9% sodium chloride, Adcocare, Adcock Ingram Critical Care) s.c. for 10 days and a 50 mg/kg intraperitoneal (i.p.) dose of ibogaine hydrochloride (HCl; Iboga Association, Cape Town, 98% purity) on day 11 (Rezvani et al., 1995; Glick et al., 1997). Ibogaine was homogenized in 1 ml/kg saline immediately before administration. The rats were terminated 24 h following the last administration.

The first morphine-ibogaine group (*n* = 10) received the escalated morphine regime for 10 days followed by a 50 mg/kg dose of ibogaine HCl on day 11. The rats were terminated 24 h following the last administration.

The second morphine-ibogaine group (*n* = 10) received the same treatment as the first morphine-ibogaine group except the rats were terminated 72 h following the last administration. The experiment was designed for morphine-ibogaine group 1 to evaluate myelination 24 h after treatment and for morphine-ibogaine group 2 to represent myelination at 72 h post treatment which has previously been found to be peak remyelination (Skripuletz et al., 2011).

Rats were decapitated with a sharp, well-maintained guillotine on the day of termination.

### 2.2 White matter tissue collection from the Sprague Dawley rat brains

Brains were carefully removed from the skulls immediately following decapitation, the white matter of the internal capsule was dissected from the brains on ice, transferred to separate 1.5 mL Eppendorf tubes and flash-frozen in liquid nitrogen prior to storage at −80°C for subsequent tissue processing.

### 2.3 White matter sample preparation for qPCR and western blotting

The frozen brain samples were homogenized with a homogenizer in QIAzol lysis reagent (Qiagen, Netherlands) to disrupt the cells. The QIAzol RNeasy Lipid Tissue Mini Kit (Qiagen, Netherlands) was used, following the manufacturer's instructions. The kit allowed for separation of the homogenate into three phases: RNA in the upper aqueous phase, DNA in the interphase and proteins in the lower organic phase. RNA was processed as per the kit's instructions.

The protein fractions from the Qiazol lysis homogenate were added to ice cold acetone in a 1:1 ratio, centrifuged at 14,500 rpm for 10 min and the supernatant was discarded. The pellet was homogenized with 5% sodium dodecyl sulfate (SDS).

### 2.4 Bicinchoninic acid assay of total proteins extracted from white matter

BCA assays are used to determine the concentration of total protein in a sample. This is done using a standard curve of Bovine Serum Albumin (BSA). The protein samples that were prepared in 5% SDS were measured by diluting them with 5% SDS to 1:5, 1:10, or 1:20 depending on their concentration as the highest limit of this standard curve was 2,000 μg/ml. The Pierce™ BCA Protein Assay kit (Thermoscientific, Massachusetts, USA) was used according to manufacturer instructions. Samples and the working reagent were added in duplicates to the plate in a 1:8 ratio of sample to working reagent. The plate was incubated at 37°C for 30 min and the absorbance read on the Biorad iMark™ microplate reader (Microplate Manager Software, Bio-Rad) at 570 nm.

### 2.5 Reverse transcription of extracted mRNA from white matter into cDNA

The Superscript™ III First Strand Synthesis system for RT-PCR (Invitrogen, Massachusetts, USA) was used to convert 2 μg RNA from each sample into cDNA, according to manufacturer's instructions. Final cDNA concentrations were measured with a Nanodrop™ _ND-1000 (Massachusetts, USA) spectrometer and diluted with RNase free water to 100 ng/μl. Aliquots were stored at 4°C.

### 2.6 qPCR protocol and data analysis

Primers are listed in Table 1 and were manufactured by Inqaba Biotec™, Pretoria, South Africa. An annealing temperature gradient test revealed that all primer sets worked efficiently at an annealing temperature of 51°C.

**Table 1:** Primers for genes used in qPCR (CNP, MBP, and beta actin).

**Gene**	**Forward primer**	**Reverse primer**
CNP (Hattori et al., 2014)	5′-CAA CAG GAT GTG GTG AGG A-3′	5′-CTG TCT TGG GTG TCA CAA AG-3′
MBP (Ray et al., 2003)	5′-TGA AAA CCC AGT AGT CCA C-3′	5′-GGA TTA AGA GAG GGT CTG C-3′
β-actin (Ray et al., 2003)	5′-TAC AAC CTC CTT GCA GCT CC-3′	5′-GGA TCT TCA TGA GGT AGT CTG TC-3′

Quantitative PCR was done in 10 μl reactions using the 2X Fast SYBR™ Green Master Mix (Thermofischer Scientific, Massachusetts, United States) with 6 μm each of the reverse and forward primers and 100 ng of cDNA per reaction. Reactions were setup in triplicate and included the corresponding beta actin housekeeping gene on the same 96-well plate. Negative, no DNA controls were also included in triplicates for both housekeeping gene and genes of interest. The Lightcycler^®^ 96 instrument and software (Roche Diagnostics, Indianapolis, United States of America) were used to run the reactions and extract the Cq values for each curve. Thermal cycling conditions were as follows: 95°C for 10 min, 45 cycles of 95°C for 15 s, 51°C for 20 s, and 72°C for 30 s. A melting curve was included at the end of all runs, with the following parameters: 95°C for 10 s, 65°C for 1 min and 97°C for 1 s.

Delta delta Cq value was used to calculate the fold change with the following formula:


$$\begin{array}{l} \Delta \Delta Cq=(Cq_{\text{gene of interest}}-Cq_{\text{β-actin}}{)}_{\text{test group}}\\ \;-(Cq_{\text{gene of interest}}-Cq_{\text{β-actin}}{)}_{\text{saline group}}\\ Fold\;change=E^{-\Delta \Delta Cq} \end{array}$$

### 2.7 SDS PAGE and western blot analysis of the white matter total protein from SD rats

Sodium dodecyl sulfate polyacrylamide gel electrophoresis was used to separate the proteins in the white matter tissue before being transferred onto a nitrocellulose membrane during western blotting.

Equal amounts of protein samples were resolved by Laemmli SDS–PAGE on a 15% Tris–glycine gel (Laemmli, 1970). Proteins resolved by SDS–PAGE were then transferred to nitrocellulose membrane using blotting buffer (50 mM Tris–HCl, 200 mM glycine, pH 8.3, containing 0.1% (w/v) SDS and 20% (v/v) methanol) at 35 V overnight at 4°C in the Hoefer™ T22 mighty small transfer tank. Membranes were blocked in TBS (0.2 M NaCl and 20 mM Tris–HCl, pH 7.4) containing 0.5% (w/v) BSA (TBS-BSA) for 1 h at room temperature. Membranes were then washed 3 × with TBS (5 min/wash).

Polyclonal mouse antibodies raised against the proteins investigated in the study were used to detect their levels of expression in each rat brain sample. Membranes were separately incubated for an hour at RT with solutions of the primary antibodies, each diluted in TBS-BSA as follows: the Anti-CNPase antibody (Abcam) was diluted 1:10000, Anti-Myelin Basic Protein antibody (Biolegend) was diluted 1:2000, and Anti-beta Actin antibody (Abcam) was diluted 1:2500. The Anti-beta actin was diluted in TBST-BSA (TBS-BSA with 0.1% Tween-20). The membranes were then washed with TBST (3 × , 10 min/wash) and incubated with goat anti-mouse IgG secondary antibody conjugated to horseradish peroxidase (Abcam) for 1 h at room temperature. The secondary antibody was diluted in TBS-BSA at 1:5000 to detect the Anti-CNP and Anti-MBP primary antibodies, and at 1:2500 to detect the Anti-β-actin primary antibody. After washing the membranes (2 × , 10 min/wash with TBST, followed by 1 × for 10 min with TBS) they were incubated with the Pierce™ ECL Western Blotting Substrate (Thermofischer Scientific) for 5 min. The blots were imaged in the ChemiDoc™ MP imaging system (Bio-Rad). and the bands analyzed for peak density using the QuantityOne™ version 4.6.9 Software Programme (Bio-Rad). The data was normalized using the following formula for all proteins:


$$\begin{array}{l} Samples\;Protein\;expression=\frac{\frac{sample\;densitometric\;measurement}{loading\;control\;from\;membrane}}{\frac{actin\;densitometric\;measurement}{loading\;control\;of\;actin\;membrane}} \end{array}$$

Each sample was analyzed by western blotting in duplicate, and the protein expression values for each replicate were averaged.

### 2.8 Statistical analysis

All statistical analysis was done using GraphPad Prism 9.5.0 for Windows (GraphPad Software). Shapiro-Wilk tests were run to test for normality. ANOVA, unpaired *t*-tests and Mann-Whitney tests were run to determine significance between the groups dependent on the normality of the data. Outliers were determined at 1% using the ROUT method. The data is represented by mean ± standard error of the mean (SEM).

## 3 Results

### 3.1 Effects of ibogaine and morphine on CNP

**Figure 1:**
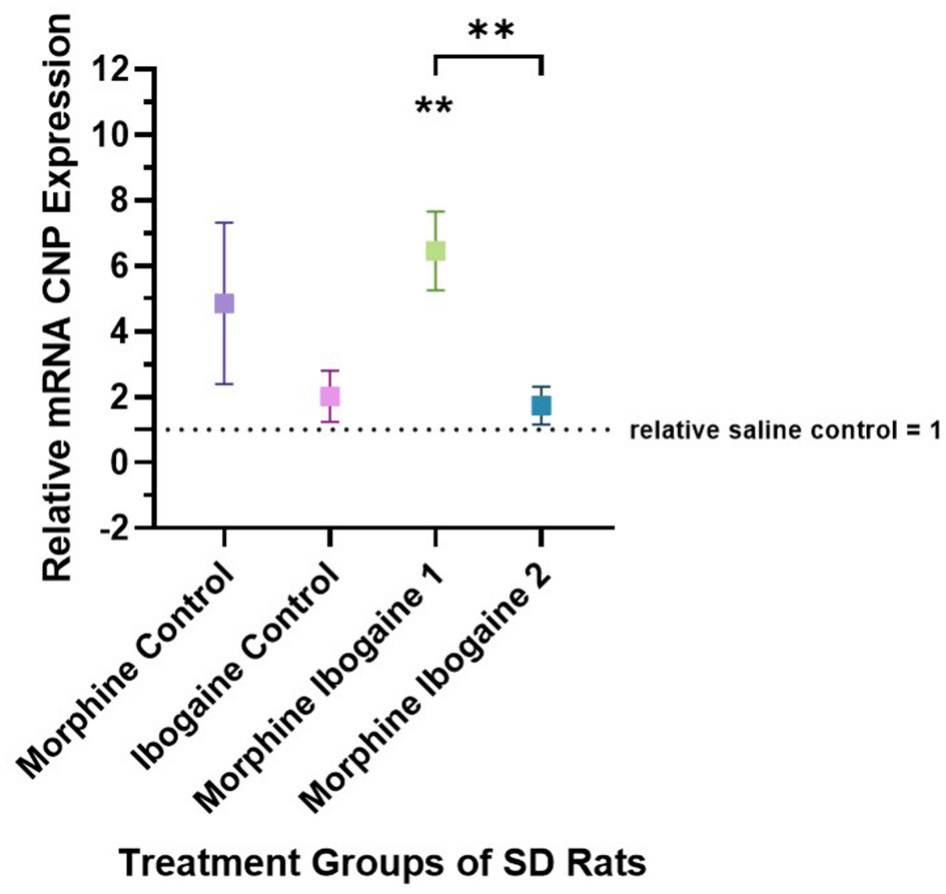
Relative mRNA expression of CNP gene in morphine control (*n* = 6), ibogaine control (*n* = 8), morphine ibogaine 1 (*n* = 8), and morphine ibogaine 2 (*n* = 7). There was an immediate increase in mRNA expression of CNP after ibogaine administration following repeated morphine administration. The data is presented as Mean ± SEM. **Shows significance (*p* < 0.0021) which is compared to saline.

Quantitative PCR was used to evaluate the changes in the mRNA expression of CNP in the white matter of the internal capsule of SD rats (Figure 1). Three outliers were found in this analysis: 1 in the morphine control, 1 in the ibogaine control and 1 in the morphine ibogaine 2 groups. These outliers were removed from the data set prior to analysis. Repeated morphine administration as well as ibogaine-only administration had no significant effect on CNP mRNA expression relative to the saline control. Although insignificant, morphine-only treatment trended toward increasing mRNA expression. Repeated morphine administration followed by ibogaine administration (morphine ibogaine 1) significantly increased CNP mRNA expression (*p* = 0.001). The morphine ibogaine 2 group showed no significant change in CNP mRNA expression relative to the saline control. This suggests that the immediate increase in CNP mRNA expression (24 h post administration) diminishes by 72 h post administration.

**Figure 2:**
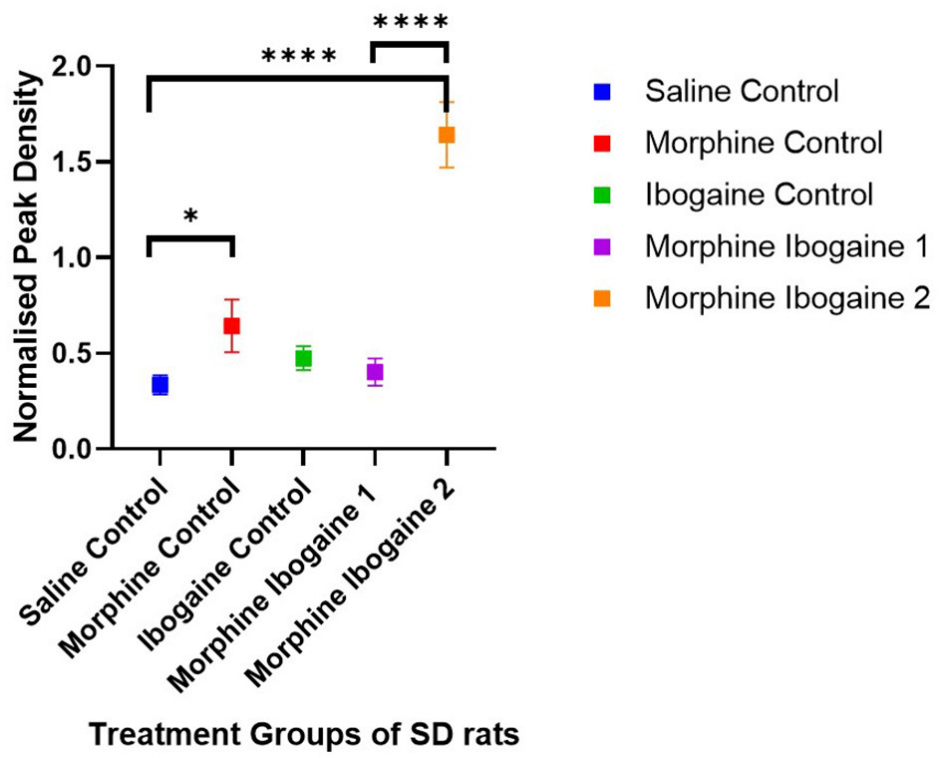
Peak density measurements of CNP protein expression of white matter in SD rats showed increased expression in morphine as well as morphine ibogaine group 2. Relative protein expression of CNP protein in saline control (*n* = 8), morphine control (*n* = 7), ibogaine control (*n* = 9), morphine ibogaine 1 (*n* = 8), and morphine-ibogaine 2 (*n* = 9). The data is presented as mean ± SEM. *Shows significance (*p* < 0.05). ****Shows significance (*p* < 0.0001).

Densitometric measurements of the western blots showed that the protein expression levels of CNPase in the white matter of SD rats increased following repeated morphine administration relative to the saline control (*p* = 0.046; Figure 2). Ibogaine administration had no effect on CNPase protein expression. Repeated morphine administration followed by ibogaine had no immediate effect on CNPase (morphine ibogaine 1), however, after 72 h (morphine ibogaine 2), there was a highly significant increase in CNPase protein expression (*p* = 0.0001).

Taken together, morphine alone upregulated CNP protein expression. Ibogaine alone had no effect on mRNA or protein expression. However, in combination, there was an early upregulation of CNP mRNA that resulted in a highly significant upregulation of CNPase protein expression after 72 h.

### 3.2 Effects of ibogaine and morphine on MBP

**Figure 3:**
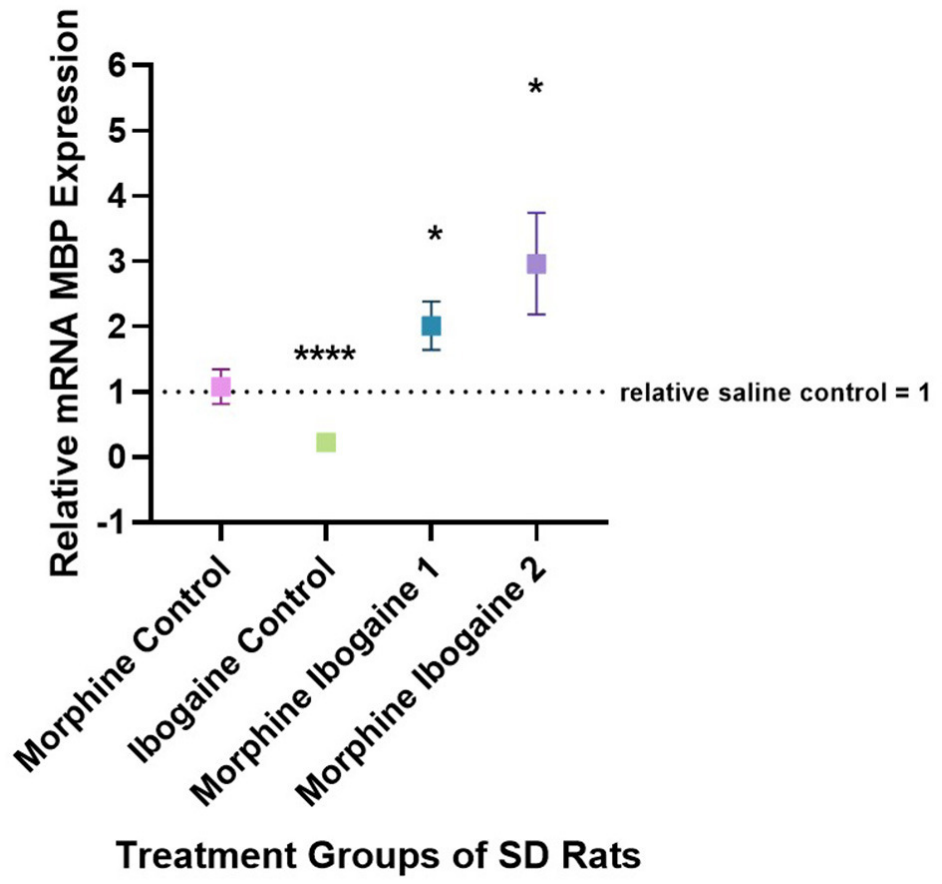
Relative mRNA expression of MBP gene in morphine control (*n* = 7), ibogaine control (*n* = 8), morphine ibogaine 1 (*n* = 8), and morphine-ibogaine 2 (*n* = 7). Relative mRNA expression of MBP is decreased after ibogaine administration and increased expression is seen in both morphine ibogaine treatment groups. The data is presented as mean ± SEM. *Shows significance (*p* < 0.5). ****Shows significance (*p* < 0.0001).

Quantitative PCR was used to evaluate the changes in the mRNA expression of MBP in the white matter of the internal capsule of SD rats (Figure 3). Two outliers were found in this analysis: 1 in the ibogaine control and 1 in the morphine ibogaine 2 groups. These outliers were removed from the data set prior to analysis. Repeated morphine administration had no effect on MBP mRNA expression relative to the saline control. Ibogaine administration significantly decreased MBP mRNA expression relative to the saline control (*p* < 0.0001). The immediate effect of ibogaine administration following repeated morphine administration (morphine ibogaine 1) was to significantly increase MBP mRNA expression relative to the saline control (*p* = 0.038) and following 72 h (morphine ibogaine 2), this increase was sustained and slightly elevated relative to the saline control (*p* = 0.041).

**Figure 4:**
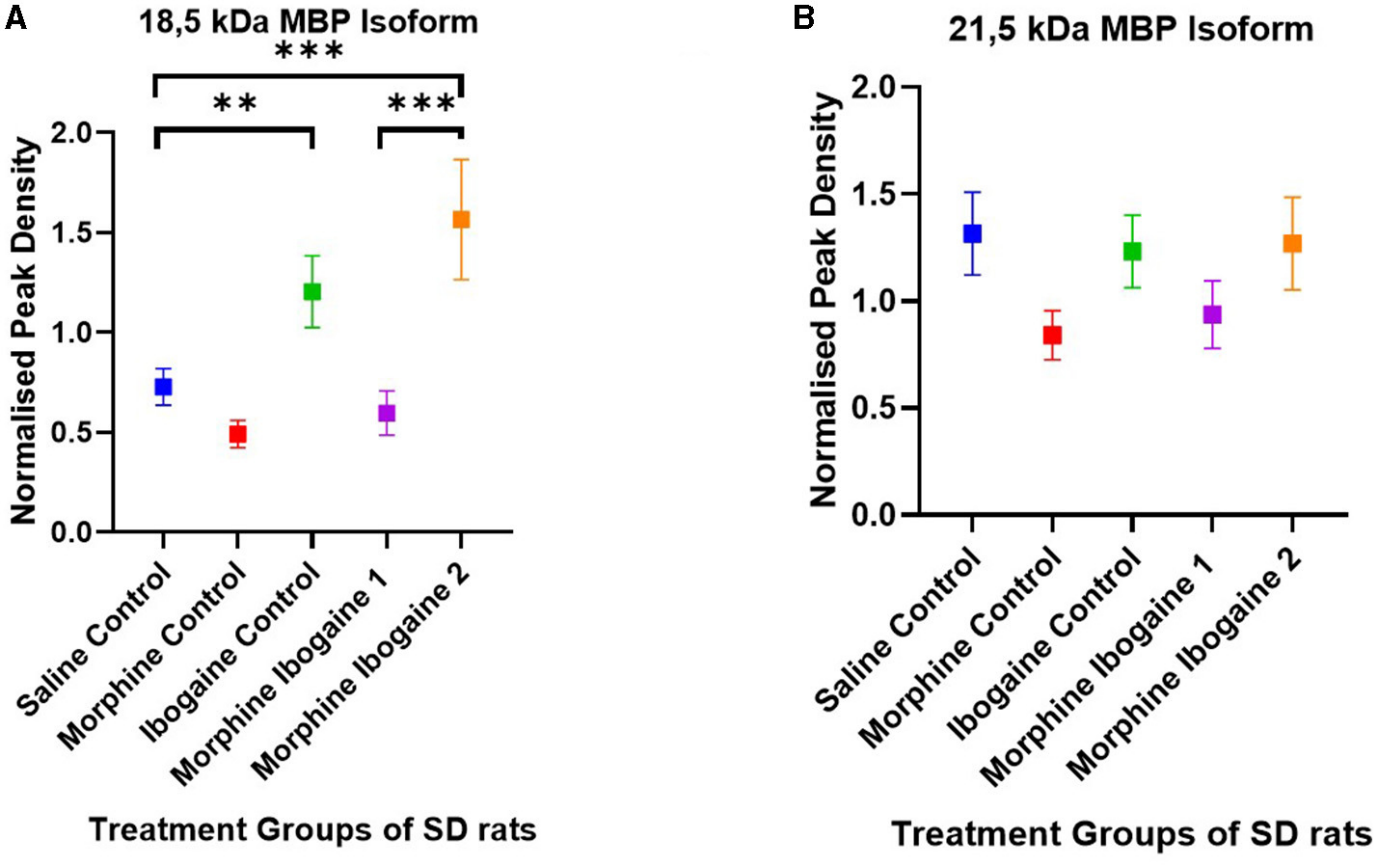
Peak density of MBP **(A)** 18.5 isoform and **(B)** 21.5 isoform in white matter of the SD rats in the saline control (*n* = 8), morphine control (*n* = 6), ibogaine control (*n* = 9), morphine ibogaine 1 (*n* = 8), and morphine-ibogaine 2 (*n* = 9). There was increased MBP expression of the 18.5 isoform after ibogaine administration and in the morphine ibogaine group 2. There was no significant differences in the peak density of the 21.5 isoform between the groups. **Shows significance (*p* < 0.0021). ***Shows significance (*p* < 0.0002).

Western blots of the white matter from the SD rats showed two isomers of MBP with molecular weights of 21.5 and 18.5 kDa (Figure 4). There were no significant differences between the test groups for the 21.5 kDa isomer (Figure 4B). The following are group differences of the 18.5 kDa isomer (Figure 4A): Repeated morphine administration had no effect on MBP protein expression relative to the saline control. Ibogaine administration significantly increased MBP protein expression relative to the saline control (*p* < 0.0021). The immediate effect of ibogaine administration following repeated morphine administration (morphine ibogaine 1) was insignificant relative to the saline control, however, following 72 h (morphine ibogaine 2), there was a highly significant increase in MBP protein expression (*p* < 0.0002) that was elevated relative to the ibogaine.

Taken together, it appears that morphine administration alone had no effect on MBP mRNA or protein expression. Ibogaine alone significantly downregulated mRNA expression yet upregulated protein expression at the same time point. In combination (repeated morphine administration followed by ibogaine administration), there was an early upregulation of MBP mRNA expression that increased after 72 h resulting in upregulated protein expression at 72 h post administration.

## 4 Discussion

Our results indicate that ibogaine alone had no effect on CNP mRNA or protein expression. Treatment with morphine-only trended toward increasing CNPase protein expression and significantly upregulated mRNA expression. In combination, ibogaine administration following repeated morphine administration had an immediate effect by significantly increasing CNP mRNA expression. This effect diminished after 72 h yet resulted in a highly significant upregulation of CNPase protein at 72 h post administration. Morphine-only treatment had no significant effect on MBP mRNA or protein expression. Ibogaine administration alone significantly upregulated protein expression yet downregulated MBP mRNA expression at the same time point. Ibogaine administration following repeated morphine administration significantly upregulated MBP mRNA expression which was elevated at 72 h post administration resulting in a highly significant upregulation of MBP protein expression at 72 h post administration.

These effects on both gene and protein expression of CNP and MBP suggest an interaction between morphine and ibogaine with regards to increasing myelination. It is also important to highlight that the interaction between morphine and ibogaine takes place after only 10 days of morphine administration. Although morphine does seem to upregulate CNPase, the combination of morphine and ibogaine augments that effect. This is shown when comparing the morphine-ibogaine groups 1 and 2 in both the mRNA and protein findings (Figures 1, 2). The mRNA is significantly upregulated 24 h post ibogaine administration (more so than the morphine control) which subsides by 72 h. The protein in morphine and ibogaine group 1 is not upregulated which, in contrast to the morphine group, confirms an interaction between morphine and ibogaine. At 72 h, CNPase shows a highly significant increase in the combination group. These findings replicate an early study we conducted where ibogaine alone had no effect on CNPase protein expression yet ibogaine administration following repeated morphine administration was significantly upregulated (Govender et al., 2020). With MBP (Figures 3, 4), the gene expression data shows a clear difference between the combination groups and the morphine- and ibogaine-only controls. The protein data is less clear but when comparing combination groups 1 and 2 in Figure 4, it is evident that combination morphine-ibogaine has less of an effect at 24 h than after 72 h. The initial mRNA upregulation results in a highly significant upregulation of the protein at 72 h which is what you would expect. Although the morphine results are unexpected and explained further in a subsequent paragraph, there is a definite augmenting effect of combination morphine and ibogaine that supersedes any substance on its own. A future experiment should include additional time points for all groups, especially morphine-only at 72 h to further clarify these findings, as this is the major limitation of the current study.

These findings also point to a possible intricate relationship between the mu and kappa opioid receptors and their roles in myelination as a possible mechanism behind the enhanced effect of the combination administration. Repeated morphine administration would downregulate the mu opioid receptor and lead to the recruitment of beta-arrestin (Valentino and Volkow, 2018). This downregulation could enhance the kappa opioid receptor mechanisms of action of ibogaine as ibogaine is a partial agonist at both kappa and mu opioid receptors. The upregulation of these myelin proteins is seen after 72 h indicating the effects are likely due to the primary metabolite, noribogaine, with a higher kapa opioid receptor affinity (Maillet et al., 2015). Dynorphin, the endogenous kappa opioid receptor ligand, is known to be released rapidly after drug administration, activating CNS and peripheral kappa opioid receptors (Khan et al., 2022). This combined with the downregulation of mu opioid receptors could enhance ibogaine's effect on the kappa opioid receptor and, thus, its involvement in myelination.

We were also able to demonstrate an early upregulation of MBP mRNA following ibogaine as well as combined morphine-ibogaine administration leading to an upregulation of MBP protein. An early upregulation of CNP mRNA in the combination morphine-ibogaine group 1 led to a significant upregulation in CNPase at 72 h post administration. This is in line with previous studies showing CNP and MBP to be the earliest markers of remyelination with the myelin proteins being detected within 3–4 days (Skripuletz et al., 2011). Our results indicate that CNP and MBP mRNA are both upregulated early yet CNP diminishes by 72 h and MBP continues to elevate. The two isomers of MBP that were seen in the western blots were 21.5 and 18.5 kDa. The 18.5 kDa isomer was increased in the ibogaine-only and morphine-ibogaine 2 group. The 21.5 kDa isoform showed no significant changes in protein expression between the test groups. These results may indicate differing activation mechanisms of the two isomers as previous research has shown the 18.5 kDa isomer to be produced later in the remyelination process than the 21.5 kDa isoform (Harauz and Boggs, 2013). Another factor that may be involved in the timing of myelination markers following ibogaine administration which needs to be explored further is the differing effects of ibogaine and noribogaine. Ibogaine is eliminated by 24 h post-administration and noribogaine takes 5–7 days to be eliminated in humans (Mash, 2023) and both are eliminated by 24 h in the rat brain (Rodriguez et al., 2020). This may influence MBP gene expression. Further studies with either additional time points, direct administration of noribogaine, or intravenous administration of ibogaine (to decrease noribogaine formation) are required to explore this fully.

Further research is needed to identify ibogaine's exact mechanisms of action on the process of myelination yet likely involve ibogaine's influence on the mTOR signaling cascade (Ly et al., 2018), production of BDNF (Marton et al., 2019), and its affinity for the kappa opioid receptor (Du et al., 2016; Mei et al., 2016). Ibogaine also has a reversible voltage gated antagonistic effect on NMDA receptors (Popik et al., 1994; Mash, 2023). The NMDA receptors present on oligodendrocytes regulate myelination and axonal health (Saab et al., 2016). NMDA receptor activation may increase myelination and CNPase by increasing the cytosolic calcium and reactive oxygen species (Cavaliere et al., 2012). Due to ibogaine's voltage gating the cytosolic calcium will need to be at lower levels to activate the NOX-dependent generation of reactive oxygen species, which will activate the PI3K/AKT pathway (Cavaliere et al., 2012). The PI3K pathway promotes neural plasticity by activating mTOR. The sigma 1 receptor agonist affinity of ibogaine could also affect the oligodendrocyte CNPase activity, as the sigma 1 receptor regulates Ca^2+^ channels, which is important for CNPase activity and regulation of gene expression (Soriani and Kourrich, 2019).

The finding of morphine upregulating CNPase and having no effect on MBP was unexpected. We initially hypothesized that repeated morphine administration would downregulate both myelination markers as opioid addiction is known to decrease white matter integrity (Bora et al., 2012; Li et al., 2013; Fan et al., 2018). Bora et al. (2012) showed that longer drug use correlated with increased white matter injury so a possible explanation could be that our administration regime was not long enough. Our administration protocol of 10 days, although adequate to facilitate neurobiological changes evident in the CNPase and combination morphine-ibogaine findings, does not represent a chronic morphine administration or opioid addiction model. It would be of great interest to the field to investigate these myelination markers in chronic morphine, conditioned-place preference or opioid self-administration models. The effects on myelination are likely to be augmented in the combination treatment groups and may show demyelination in the morphine-only groups. A recent study has shown morphine's effects on OPCs to be region specific whereby morphine-induced oligodendrogenesis occured specifically in the ventral tegmental area (Yalçin et al., 2024). Future research should, therefore, include additional white matter regions. These findings may also explain our differing results between morphine's effects on CNP vs. MBP. CNPase is an oligodendrocyte-related protein and MBP is only related to myelin which may be the reason we only see an increase in the morphine control with CNPase and not MBP. Maladaptive myelination in reward circuitry is propped by Yalçin et al. (2024) to represent a key neural substrate of pathological learning associated with OUD, suggesting myelination as a potential therapeutic target. This highlights the importance of ibogaine's role in remyelination.

There was a striking difference between ibogaine's effect on MBP protein vs. gene expression. The correlation between protein and mRNA expression is understudied with many conflicting results (Guo et al., 2008) and transcription factors would play a major role in the opposing findings. Sox2 has been identified as a crucial transcription factor for remyelination as it regulates OPC proliferation (Zhao et al., 2015; Zhang et al., 2018). Sox2 regulates OPC proliferation by increasing proliferation of the OPCs and inhibits their differentiation into mature oligodendrocytes (Zhao et al., 2015; Zhang et al., 2018). This would be essential for remyelination to occur. The transcription factors Olig1 and Olig2 have also been linked to remyelination (Wegener et al., 2015; Yang et al., 2017; Zhao et al., 2019). Another major transcription factor that is required for efficient remyelination and oligodendrocyte regeneration is Stat3 (Steelman et al., 2016). These factors should be investigated following ibogaine administration to fully understand these molecular relationships.

These findings indicate that ibogaine is able to upregulate genes and proteins involved in the process of remyelination and highlights an important mechanism of action of ibogaine's ability to repair brain injury (Cherian et al., 2024) and treat substance use disorders (Barsuglia et al., 2018; Mash et al., 2018; Mash, 2023). Future research should address ibogaine and noribogaine's effects on the mu and kappa opioid receptors as there is still disagreement in the literature regarding specific receptor affinities (Cameron et al., 2021; Mash, 2023). Experiments should be designed with chronic morphine and imaging protocols to uncover the full extent of the interaction between morphine and ibogaine and structural plasticity of white matter. These experiments may uncover ibogaine's mechanism of action in being able to treat opioid use disorders yet are also relevant for the numerous myelin-associated diseases such as stroke and multiple sclerosis.

## Data availability statement

The raw data supporting the conclusions of this article will be made available by the authors, without undue reservation.

## Ethics statement

The animal study was approved by Animal Research Ethics Committee at the University of the Witwatersrand. The study was conducted in accordance with the local legislation and institutional requirements.

## Author contributions

DG: Formal analysis, Investigation, Methodology, Writing – original draft. LM: Investigation, Methodology, Writing – review & editing. MP: Resources, Writing – review & editing. NT: Formal analysis, Methodology, Supervision, Writing – review & editing. GO: Formal analysis, Methodology, Supervision, Writing – review & editing. TC: Conceptualization, Formal analysis, Funding acquisition, Investigation, Methodology, Project administration, Supervision, Writing – review & editing.
